# Soil bacterial community response to continuous cropping of cotton

**DOI:** 10.3389/fmicb.2023.1125564

**Published:** 2023-01-26

**Authors:** Zheng Ma, Peng Li, Chuanzhen Yang, Zili Feng, Hongjie Feng, Yalin Zhang, Lihong Zhao, Jinglong Zhou, Heqin Zhu, Feng Wei

**Affiliations:** ^1^National Engineering Research Center of Cotton Biology Breeding and Industrial Technology, Institute of Cotton Research of Chinese Academy of Agricultural Sciences, Anyang, China; ^2^Institute of Cotton Research of Chinese Academy of Agricultural Sciences, Anyang, China; ^3^Zhengzhou Research Base, State Key Laboratory of Cotton Biology, Zhengzhou University, Zhengzhou, China

**Keywords:** cotton, continuous cropping obstacle, Verticillium wilt, soil microbe, amplicon sequencing

## Abstract

**Introduction:**

Long-term continuous cropping may result in the outbreak and proliferation of soil-borne diseases, as well as reduction in annual crop production. Overcoming the obstacles of continuous cropping is critical for the long-term growth of modern agriculture. Soil microbes are essential for plant health, but the consequences of continuous cropping on soil microbiome are still poorly understood.

**Methods:**

This study analyzed changes in soil bacterial community composition of Aksu (AKS) and Shihezi (SHZ) in Xinjiang Province during 1–20  years of continuous cropping by 16S amplicon sequencing. The results showed that the incidence of cotton Verticillium wilt rose with the number of cropping years. The bacterial alpha diversity in the AKS soil grew as the number of continuous cropping years increased, however it declined in the SHZ soil.

**Results:**

The results of beta diversity analysis showed that there were significant differences in soil bacterial communities between different continuous cropping years and between different soils. The results of community composition changes at the level of main phyla and genus showed that the relative abundance of Actinobacteria, Bacteroidetes and *Streptomyces* decreased with the increase of continuous cropping years in the AKS and the SHZ soils. In addition, Actinobacteria, Propionibacteriales, and Nocardioidaceae were significantly enriched during the early stages of continuous cropping. Network analysis showed that long-term (≥8  years) continuous cropping interfered with the complexity of soil bacterial co-occurrence networks and reduced collaboration between OTUs.

**Discussion:**

These findings suggested that continuous cropping and soil origin jointly affected the diversity and structural of bacterial communities, and the loss of Nocardioidaceae and *Streptomyces* in Actinobacteria might be one of the reasons of continuous cropping obstacles.

## Introduction

The region of Xinjiang is China’s most significant cotton-growing site (Appiah et al., 2014; Feng et al., 2017), owing to a combination of climatic characteristics such as long hours of sunlight, dry air, and low annual precipitation that are ideal for cotton cultivation (Wei and Yu, 2018, 2019). In Xinjiang, 60–70% of the cultivated land is planted with cotton, of which more than 95% are in some major producing areas (Yang et al., 2018). As a result, continuous cropping is prevalent in the region, and cotton has been continuously planted in some cultivated lands for more than 20 years. However, long-term continuous cropping can lead to a decline in cotton yield and quality, and an increase in the incidence of Verticillium wilt, which seriously hampers the development of the cotton industry (Feng et al., 2003; Han et al., 2017; Xi et al., 2019).

Overcoming continuous cropping obstacles is an urgent task for the sustainable development of modern agriculture. According to research, changes in soil microbial populations are one of the primary drivers of continuous cropping obstacles (Van Elsas et al., 2002; Zhang et al., 2013). The soil microbiome is essential for soil health, since it participates in the biogeochemical cycle, regulates nutrients and soil-borne diseases, and improves soil functioning (Van Der Heijden et al., 2008; Dai et al., 2020). These microbial community functions can maintain the sustainability of soil health and in turn enhance crop productivity (Ashworth et al., 2017). Therefore, a diverse soil microbial community is essential for soil health. Soil microbial community composition has been shown to be influenced by various factors, such as agricultural management, soil type, organic amendments, and plant species (Van Elsas et al., 2002; Garbeva et al., 2004; Qiu et al., 2012).

Recently, increasing numbers of studies have indicated that continuous cropping resulted in the disruption of soil microbial community and structure. For example, Xiong et al. (2015b) evaluated the soil bacterial community in vanilla continuous cropping time-series fields, and found that the relative abundance of Firmicutes, Actinobacteria, and Bacteroidetes declined as the number of continuous cropping years increased. Wei and Yu (2018) investigated the composition of the soil bacterial community under long-term continuous cotton planting, and found that continuous cotton cropping increased the alpha diversity of bacterial communities, with Firmicutes, Actinobacteria, and Acidobacteria populations changing significantly. Although previous studies provided some information on the effects of continuous cropping on soil microbes, there are few studies comparing the effects of continuous cropping on soil microbial communities in different soils. Therefore, it is necessary to systematically study the impact of continuous cotton cropping on soil microecology.

Cotton Verticillium wilt, caused by the soil-borne plant pathogenic fungus *Verticillium dahliae*, is one of the worst plant diseases. *V. dahliae* can survive as microsclerotia in soil without host plants for more than 14 years. *V. dahliae* is exceedingly difficult to control due to its long survival time in soil, strong variability, and complex infection process of cotton (Klosterman et al., 2009). Soil fumigation using chemical agents is effective in controlling soil-borne pathogens, but some fumigants have been banned by legislation (Martin, 2003). The use of beneficial microbes to prevent and control plant diseases is friendly to the ecological environment and has broad application prospects. Therefore, studying the impact of continuous cropping on soil microbiome is helpful to seek biological control strategies for plant diseases.

In this study, 16S amplicon sequencing technology was used to analyze and compare the changes of soil bacterial communities in cotton fields in the Aksu (AKS) region of southern Xinjiang and the Shihezi (SHZ) region of northern Xinjiang over a period of 1–20 years. This study aimed to investigate the response of soil microbial community to continuous cropping, which would provide useful guidance for improving continuous cropping obstacles using microbial techniques in the future.

## Materials and methods

### Site description and soil sampling

The two study sites are located in Aksu and Shihezi, Xinjiang, China. Aksu belongs to the temperate continental arid climate; the annual average temperature is between 9.2 and 11.5°C, the annual average total sunshine hours are between 2,571 and 2,967 h, and the annual frost-free period is between 183 and 250 days; the annual average rainfall is only 64 mm, while the annual evaporation amounted to 1,890 mm; the distribution of rainfall within the year was extremely uneven, with the main rainfall distributed in summer and almost no rainfall in winter (Yang et al., 2021). Shihezi has a continental arid climate with annual average temperature range between 7.5 and 8.2°C, sunshine duration between 2,318 and 2,732 h, frostless period of 147–191 days; the annual evaporation (range between 1,000 and 1,500 mm) is about 6 times of annual precipitation (range between 180 and 270 mm) (Zhu et al., 2022).

A total of 14 treatments were conducted in this experiment: the farmland with continuous planting of cotton for 1, 3, 5, 8, 10, 15, and 20 years in two sites (Aksu and Shihezi), named AKS1, AKS3, AKS5, AKS8, AKS10, AKS15, AKS20, SHZ1, SHZ3, SHZ5, SHZ8, SHZ10, SHZ15, and SHZ20, respectively. Two cotton fields were selected in each continuous cropping year at each location. There were three blocks in each cotton field and six blocks in each treatment. At the boll formation stage (late August 2020), the incidence of cotton Verticillium wilt was investigated and the incidence rate was calculated (incidence rate = number of diseased plants/total number of investigated plants × 100%). Soil samples were collected with a sampler (2.5 cm in diameter, 0–20 cm deep) using the five-point sampling method, and five soil cores collected in each block were pooled into one sample, with 6 mixed samples per treatment. A total of 84 soil samples were collected. Prior to DNA extraction, the obtained soil was sieved (2 mm) and stored in a refrigerator at −80°C.

### Soil DNA extraction and amplicon sequencing

Soil samples (250 mg) were resuspended in 500 μL of MoBio PowerSoil bead solution, and total genomic DNA was extracted from all 84 soil samples using the MoBio PowerSoil DNA Isolation Kit (MoBio Laboratories, Carlsbad, CA, United States) following the manufacturer’s instructions. Successful DNA extraction was checked on a 1% agarose gel and DNA concentration was assessed using a spectrophotometer NanoDrop ND-2000 (NanoDrop Technologies, Wilmington, DE, United States). Barcode primers 341F (5′-CCTAYGGGRBGCASCAG-3′) and 806R (5′-GGACTACNNGGGTATCTAAT-3′) were used to amplify the V3-V4 hypervariable region of 16S rRNA. PCR reactions were performed in a 30 μL reaction mix containing 15 μL Phusion^®^ High-Fidelity PCR Master Mix (New England Biolabs), 0.2 μM forward primer, 0.2 μM reverse primer, and 10 ng template DNA (Wei et al., 2019). PCR conditions were as follows: 98°C for 1 min; 30 cycles of 98°C for 10 s, 50°C for 30 s, 72°C for 30 s; and a final 72°C extension for 5 min. The PCR products were confirmed by electrophoresis on a 2% agarose gel and purified using the GeneJET Gel Extraction Kit (Thermo Scientific, Fermentas, United States). The TruSeq^®^ DNA PCR-Free Sample Preparation Kit (Illumina, San Diego, CA, United States) was used to generate sequencing libraries, and the quality of each library was assessed using Qubit 2.0 Fluorometer (Life Technologies, United States). Finally, the library was sequenced using an Illumina HiSeq 2500 and 250 nucleotide paired-end reads were generated.

### Sequencing data analysis

Paired-end reads were assembled using FLASH (v1.2.7) (Magoč and Salzberg, 2011). Quality filtering was performed according to the QIIME (v1.7.0) quality control process (Caporaso et al., 2010; Bokulich et al., 2013). After removal of chimeras, the remaining high-quality sequences were clustered into operational taxonomic units (OTUs) with 97% sequence similarity using Uparse software (v7.0.1001) (Edgar, 2013). Each representative sequence was analyzed for species annotation using the bacterial database SILVA 138 (Quast et al., 2013).

### Bioinformatics and statistical analysis

Community alpha diversity was evaluated by Chao1 estimator and Shannon diversity index calculated by QIIME, and visualized using R software (v3.5.0). The alpha diversity index among the groups was statistically analyzed using one-way analysis of variance (ANOVA) and Tukey’s multiple comparison test (*p* < 0.05 was considered significant) in SPSS 22.0. Beta diversity analysis was performed based on the Bray–Curtis dissimilarity distance matrix and visualized by principal coordinate analysis (PCoA). Analyzes of similarity (ANOSIM) were performed using the vegan package in R to identify significant differences in beta diversity across groups. The effects of continuous cropping years, soil origin and their interactions on bacterial alpha diversity and beta diversity were evaluated through the two-way ANOVA using SPSS 20.0. Linear discriminant analysis (LDA) effect size (LEfSe) was performed on the Galaxy platform^1^ to identify the most differentially abundant taxa biomarker between continuous cropping years (LDA score > 4.0). Co-occurrence network analysis was performed using the iNAP platform^2^ (Feng et al., 2022). The OTUs were used to construct networks across the entire soil for 1, 3, 5, and 8 years of continuous cropping (short-term continuous cropping, ST), and for 8, 10, 15, and 20 years of continuous cropping (long-term continuous cropping, LT), respectively. Spearman correlation analysis was performed on OTUs with mean relative abundance >0.05%, all pairwise associations with |*r*| > 0.8 and *p* < 0.05 were retained and visualized using ChiPlot.^3^

## Results

### Field disease development

Overall, the incidence of cotton Verticillium wilt in AKS and SHZ cotton fields rose as the number of continuous cropping years increased. Incidence of cotton Verticillium wilt was 2.55% in the AKS cotton field with continuous cropping for 1 year and 66.41% in the AKS cotton field with continuous cropping for 20 years (Supplementary Figure 1). Cotton Verticillium wilt incidence was 0% in the SHZ cotton field with continuous cropping for 1 year and 63.27% in the SHZ cotton field with continuous cropping for 20 years. Two-way ANOVA showed that the incidence of cotton Verticillium wilt was significantly affected by the continuous cropping years and soil origin as well as their interactions (Table 1).

**Table 1:** Two-way ANOVA for Verticillium wilt incidence and soil bacterial community properties as affected by continuous cropping years (year), soil origin (soil) and the interaction (year  ×  soil).

Factor	Incidence		Chao1		Shannon		PCo1		PCo2	
	F	P	F	P	F	P	F	P	F	P
Year	556.263	**<0.001**	12.882	**<0.001**	33.281	**<0.001**	24.311	**<0.001**	12.666	**<0.001**
Soil	29.632	**<0.001**	82.788	**<0.001**	191.79	**<0.001**	164.255	**<0.001**	428.727	**<0.001**
Year × Soil	14.325	**<0.001**	11.528	**<0.001**	38.553	**<0.001**	29.166	**<0.001**	25.781	**<0.001**

The categorical factors are continuous cropping years (1, 3, 5, 8, 10, 15, 20), soil origin (AKS, SHZ). Incidence, incidence of cotton Verticillium wilt; Chao1, Chao1 estimator; Shannon, Shannon diversity index; PCo1, first principal coordinate in PCoA; PCo2, second principal coordinate in PCoA. Statistically significant values are in bold (*p* < 0.05).

### Sequence data and alpha diversity

**Figure 1:**
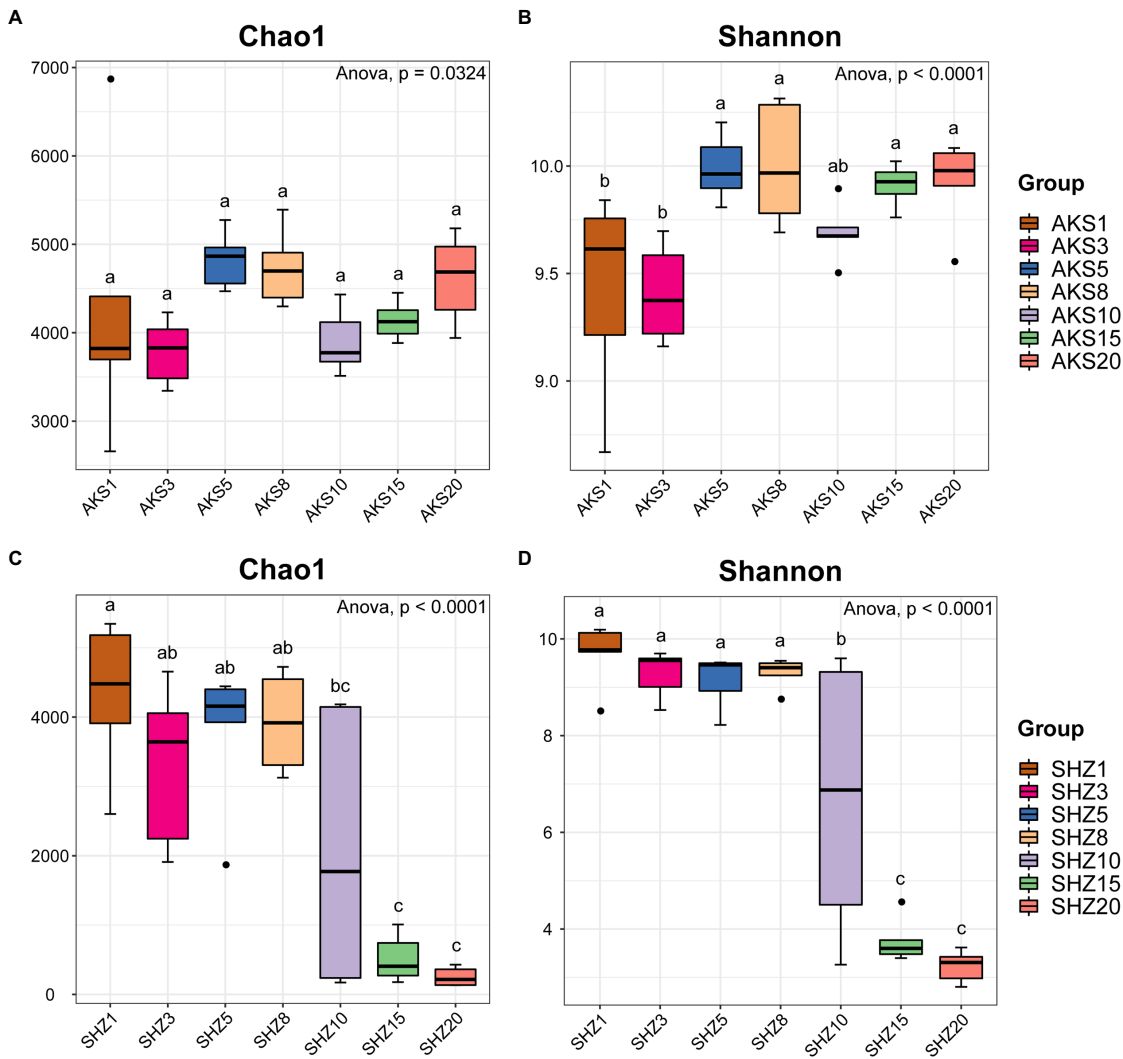
Chao1 index **(A,C)** and Shannon index **(B,D)** of cotton field soil samples bacterial community with different continuous cropping years in AKS and SHZ soils. AKS1, AKS3, AKS5, AKS8, AKS10, AKS15, and AKS20: AKS soils of continuous cropping 1, 3, 5, 8, 10, 15, and 20 years; SHZ1, SHZ3, SHZ5, SHZ8, SHZ10, SHZ15, and SHZ20: SHZ soils of continuous cropping 1, 3, 5, 8, 10, 15, and 20  years. The letters ‘a’, ‘b,’ and ‘c’ represent statistically significant differences between different treatments (*p* < 0.05, Tukey test).

The raw sequence count for the 84 samples varied from 73,459 to 109,958, with an average of 99,760. The effective tags acquired following quality control and the elimination of chimeras varied between 33,145 and 77,792, with an average of 57,116. The sequences were clustered into OTUs with 97% concordance, yielding 13,657 OTUs in total. The annotation results showed that a total of 3,839 OTUs (28.11%) were annotated to the genus level. The dilution curves demonstrated that the sequencing data accurately represented the diversity present in the samples from each treatment group (Supplementary Figure 2). Alpha diversity analysis revealed significant differences in the Chao1 and Shannon indices between cotton field samples with different continuous cropping years in AKS and SHZ soils (*p* < 0.05) (Figure 1). In the AKS soil, the Shannon index of 1 and 3 years of continuous cropping was significantly lower than that of 5, 8, 15, and 20 years of continuous cropping (*p* < 0.05) (Figure 1B). However, in the SHZ soil, the Chao1 index of 1, 3, 5, and 8 years of continuous cropping were significantly higher than those of 15 and 20 years of continuous cropping (*p* < 0.001) (Figure 1C); the Shannon index of 1, 3, 5, and 8 years of continuous cropping were significantly higher than those of 10, 15, and 20 years of continuous cropping (*p* < 0.01) (Figure 1D); the Shannon index of 10 and 15 years of continuous cropping was significantly higher than that of 20 years (*p* < 0.001). Two-way ANOVA showed that alpha diversity indexes (Chao1 and Shannon) were significantly affected by the continuous cropping years and soil origin as well as their interactions (Table 1).

### Beta diversity: PCoA

**Figure 2:**
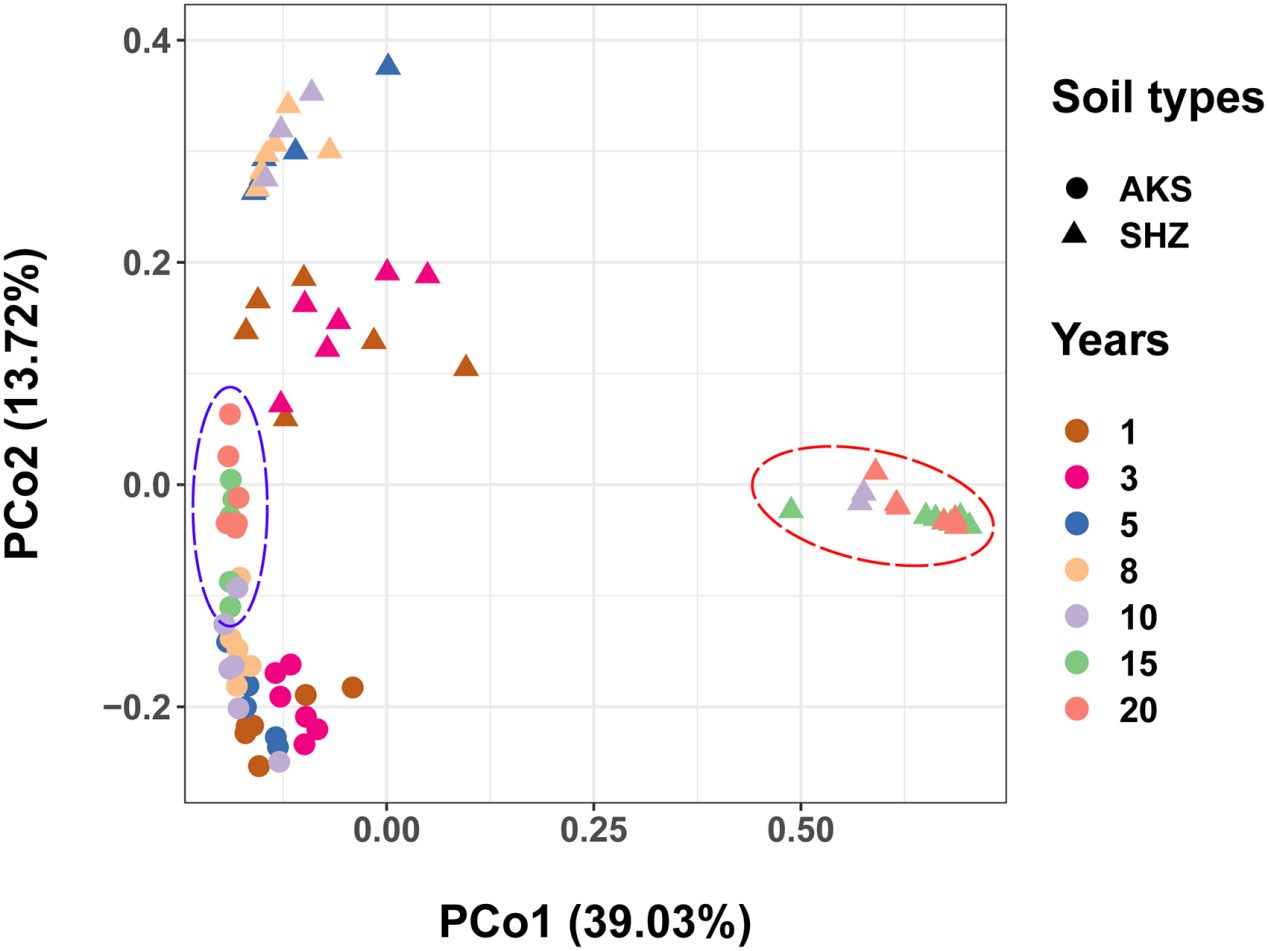
Principal coordinate analysis (PCoA) based on Bray-Curtis distance for the bacterial community structure of cotton field soil samples with different continuous cropping years in AKS and SHZ soils.

PCoA analyses using Bray-Curtis distances demonstrated that rhizosphere samples from the same treatment grouped together (Figure 2). The analysis of similarities (ANOSIM) showed that there were significant differences in community structure between different treatments (*R* = 0.6621, *p* = 0.001), and there were also significant differences in community structure between AKS and SHZ soils (*R* = 0.4545, *p* = 0.001). In both soils, the distance between samples of continuous cropping for 1 and 3 years was closer, and the distance between the samples of continuous cropping for 5 and 8 years was closer. In particular, the samples of continuous cropping for 15 and 20 years clustered together and separated from the samples of other continuous cropping years. Two-way ANOVA showed that beta diversity indexes (PCo1 and PCo2) were significantly affected by the continuous cropping years and soil origin as well as their interactions (Table 1).

### Overview of species composition at the phylum level

**Figure 3:**
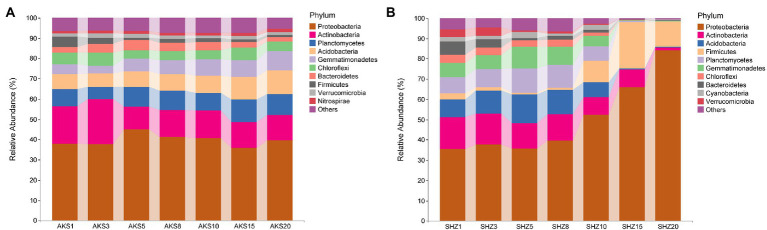
The relative abundance of the top 10 bacterial phyla in cotton field soil samples with different continuous cropping years in AKS **(A)** and SHZ **(B)** soils.

Bacterial sequences from all samples were classified into 44 phyla. Proteobacteria, Actinobacteria, Planctomycetes, Acidobacteria, Gemmatimonadetes, and Chloroflexi were the dominating bacterial phyla in the AKS soil samples, accounting for more than 80% of all sequences (Figure 3A). On the other hand, Proteobacteria, Actinobacteria, Acidobacteria, Firmicutes, Planctomycetes, and Gemmatimonadetes were the predominant bacterial phyla in the SHZ soil samples, accounting for more than 75% of all sequences (Figure 3B). In the top 10 phyla of AKS soil, with the increase of continuous cropping years, the relative abundance of Planctomycetes, Acidobacteria, Gemmatimonadetes and Nitrospirae gradually increased, while the relative abundance of Actinobacteria and Firmicutes gradually decreased; the relative abundance of Bacteroidetes increased gradually in the first 5 years of continuous cropping and then decreased gradually. In the top 10 phyla of SHZ soil, the relative abundance of Proteobacteria and Firmicutes increased significantly after 10 years of continuous cropping, while the relative abundance of other phyla decreased significantly.

### Overview of species composition at the genus level

**Figure 4:**
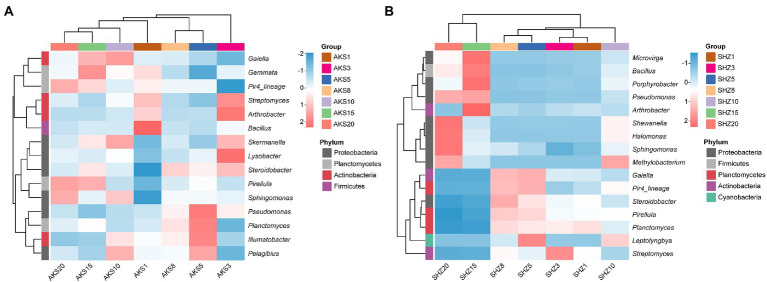
Composition of high-abundance bacterial genera (relative abundance >0.5%) in cotton field soil samples with different continuous cropping years in AKS **(A)** and SHZ **(B)** soils.

The bacterial sequences of all samples were categorized into 781 different genera. Bacterial genera having a relative abundance larger than 0.5% in both soil datasets were further evaluated (Figure 4). The clustering tree showed that the abundance of bacterial genera with continuous cropping for 5 and 8 years had high similarity, and they also clustered in the same cluster with continuous cropping for 15 and 20 years. In the AKS soil, the relative abundance of *Sphingomonas* and *Pirellula* increased with the continuous cropping years, while the relative abundance of *Arthrobacter*, *Streptomyces* and *Bacillus* decreased; the relative abundance of *Pseudomonas* gradually increased in the first 5 years of continuous cropping, and then gradually decreased. In the SHZ soil, with the increase of continuous cropping years, the relative abundance of *Sphingomonas*, *Shewanella*, and *Methylobacterium* gradually increased, while the relative abundance of *Planctomyces* and *Streptomyces* decreased gradually; the relative abundances of *Bacillus*, *Pseudomonas*, *Halomonas*, *Microvirga*, and *Porphyrobacter* gradually decreased in the first 8 years of continuous cropping, and then gradually increased; the relative abundances of *Pirellula* and *Steroidobacter* gradually increased in the first 8 years of continuous cropping, and then gradually decreased.

### Variation in bacterial taxa abundances

**Figure 5:**
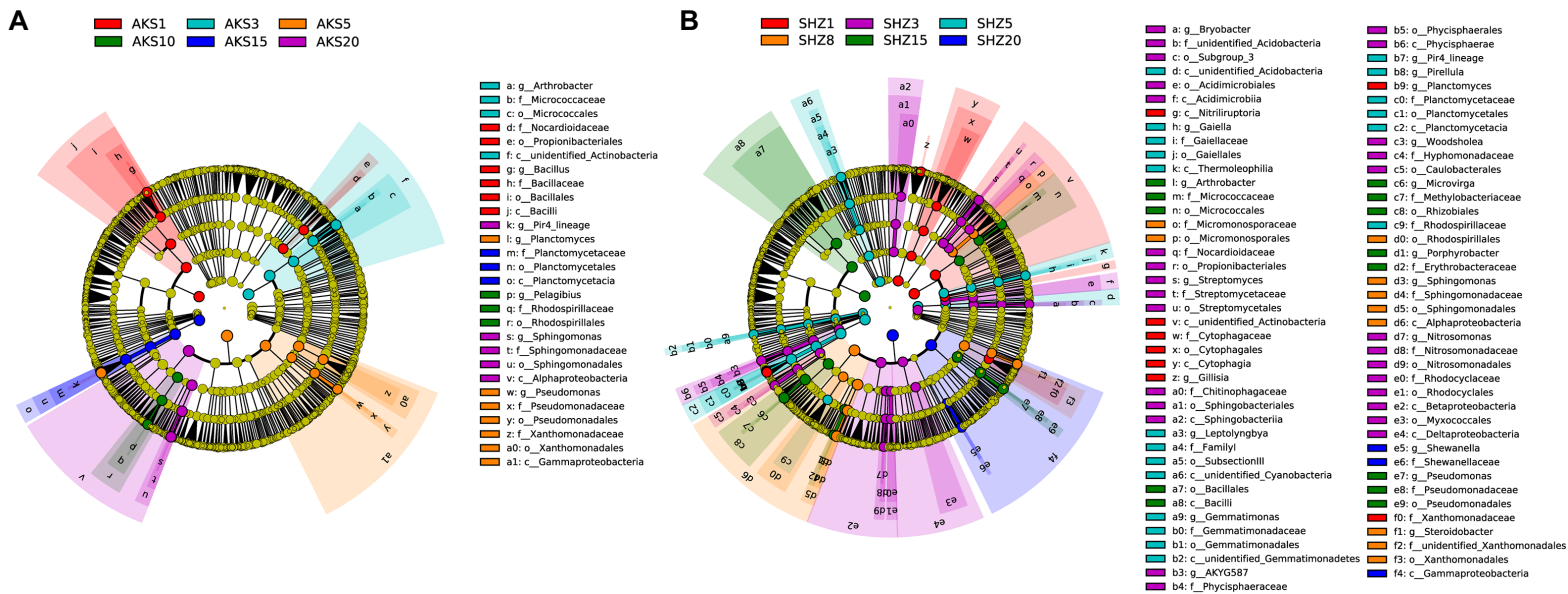
Cladograms of cotton field soil samples bacterial taxa with different continuous cropping years in AKS **(A)** and SHZ **(B)** soils based on linear discriminant analysis effect size (LEfSe).

The linear discriminant analysis (LDA) effect size (LEfSe) method was used to analyze the bacterial taxa with significant abundance difference between different continuous cropping years in two soils. A total of 28 taxa were found to be significantly different in relative abundance among continuous cropping years in the AKS soils (Figure 5A and Supplementary Figure 3), and the corresponding value was 81 in the SHZ soils (Figure 5B and Supplementary Figure 4). Propionibacteriales, Nocardioidaceae, and Actinobacteria were significantly enriched in both soils with continuous cropping for 1–3 years. Firmicutes, Bacilli and Bacillales were significantly enriched in the AKS soil of 1 year of continuous cropping and the SHZ soil of 15 years of continuous cropping, respectively. Micrococcales, Micrococcaceae, and *Arthrobacter* were significantly enriched in the AKS soil of 3 years of continuous cropping and the SHZ soil of 15 years of continuous cropping, respectively. Pseudomonadales, Pseudomonadaceae, and *Pseudomonas* were significantly enriched in the AKS soil of 5 years of continuous cropping and the SHZ soil of 15 years of continuous cropping, respectively. Proteobacteria and Gammaproteobacteria were significantly enriched in the AKS soil of 5 years of continuous cropping and the SHZ soil of 20 years of continuous cropping, respectively. Planctomycetes, Planctomycetales, Planctomycetaceae, and Planctomycetacia were significantly enriched in the AKS soil of 15 years of continuous cropping and the SHZ soil of 5 years of continuous cropping, respectively.

### Co-occurrence network

**Figure 6:**
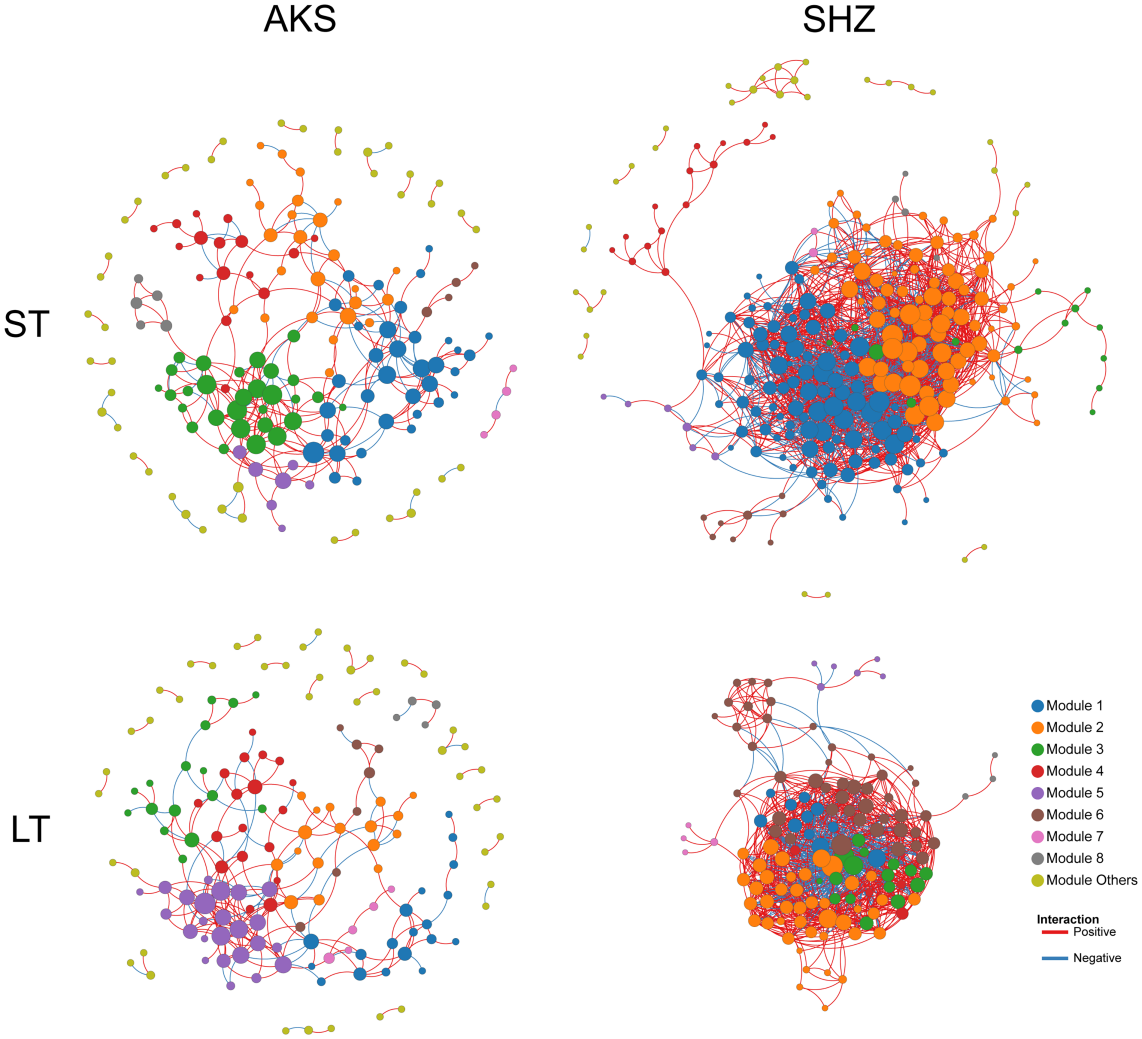
Co-occurrence networks of bacterial communities in cotton field soil samples under short-term continuous cropping (ST) and long-term continuous cropping (LT) in AKS and SHZ soils. ST is the sample summary of 1, 3, 5, and 8 years of continuous cropping. LT is the sample summary of 8, 10, 15, and 20 years of continuous cropping.

Co-occurrence networks were constructed to evaluate the co-occurrence patterns of bacterial communities in short-term and long-term continuous cropping in the two soils. In the AKS soil, compared with short-term (≤8 years) continuous cropping, long-term (≥8 years) continuous cropping decreased the network total nodes, total links, average degree, average clustering coefficient and density, while the average path distance increased (Figure 6 and Table 2). In the SHZ soil, compared with short-term continuous cropping, long-term continuous cropping decreased network total nodes, total links and average path distance, but increased average degree, average clustering coefficient and density (Figure 6 and Table 2). Interestingly, the ratio of positive and negative connections in the network of long-term continuous cropping was less than that of short-term continuous cropping in both soils (Table 2).

**Table 2:** Topological properties of the four co-occurrence networks in Figure 6.

	AKS soil		SHZ soil	
	ST	LT	ST	LT
Total nodes	169	154	256	130
Total links	312	239	2,091	1,163
Positive links	247	182	1,473	791
Negative links	65	57	618	372
Positive links/Negative links	3.8	3.193	2.383	2.126
Average degree	3.692	3.104	16.336	17.892
Average clustering coefficient	0.243	0.187	0.409	0.447
Average path distance	4.6	4.822	3.244	2.607
Density	0.022	0.02	0.064	0.138

## Discussion

In this study, the incidence of Verticillium wilt in cotton field soil with different continuous cropping years was assessed in AKS and SHZ, and it was found that the incidence of Verticillium wilt rose with the continuous cropping years, especially after 5 years of continuous cropping. Similar phenomena have been observed in many crops, including cucumber, potato, tomato and vanilla, with an increased incidence of soil-borne diseases and severely stunted growth after years of continuous cropping (Zhou and Wu, 2012; Lu et al., 2013; Li et al., 2014; Xiong et al., 2015b).

Microbial diversity and community structure are important indicators affecting soil health, and many microbes have the potential to improve crop yield and disease suppression (Ferris and Tuomisto, 2015; Pereg and McMillan, 2015). This study systematically evaluated the composition of soil bacterial communities in the AKS and the SHZ soil of cotton fields continuously cropping for many years, and revealed the mechanism of continuous cropping obstacles from the soil microbial level. The results showed that alpha diversity of bacterial communities with different continuous cropping years had different changing trends in two soils. In the AKS soil, bacterial alpha diversity rose with increasing cropping years, except for a decrease at 10 years of continuous cropping. Previous studies have also reported that continuous cotton cultivation leads to increased soil bacterial diversity (Wei and Yu, 2018). However, in the SHZ soil, alpha diversity of bacteria declined as the number of cropping years increased, especially after 10 years of continuous cropping. Previous studies showed that soil bacterial diversity decreased with continuous cropping time under continuous planting of cotton, potato, and black pepper (Liu et al., 2014; Xiong et al., 2015a; Yang et al., 2018). The results of beta diversity analysis showed that there were significant differences in soil bacterial communities of different continuous cropping years, especially the samples of 15 and 20 years continuous cropping were far from those of other years; meanwhile, the samples between the two soils were separated from each other. These results indicated that continuous cropping altered soil bacterial diversity and community structure. Although the agricultural management measures of cotton fields in the aforementioned two regions were similar, the alpha diversity and structural composition of soil bacterial communities exhibited different changing trends under continuous cropping, which might be mainly attributable to soil properties. Two-way ANOVA confirmed that the alpha diversity and beta diversity of bacteria were affected by continuous cropping years, soil origin and their interaction. The effects of soil types with a range of factors including structure, texture, pH, water content, salinity, mineral content, and organic content on soil microbial communities have been widely demonstrated (Berg and Smalla, 2009; Bach et al., 2010; O’Brien et al., 2019).

At the phylum level of soil bacteria, Proteobacteria, Actinobacteria, Acidobacteria, Planctomycetes, and Firmicutes were the predominant bacterial phyla in the soil of continuous cropping cotton, with Proteobacteria being the phylum with the highest proportion, which was consistent with previous findings (Tian and Gao, 2014; Tian et al., 2020). Proteobacteria are involved in the global carbon, nitrogen, sulfur, and iron cycles and are therefore important for maintaining soil microbial community homeostasis (Spain et al., 2009; Shin et al., 2015). We found that the relative abundance of Actinobacteria and Bacteroidetes in the AKS and the SHZ soils decreased with the increase of continuous cropping years. It has been reported that Actinobacteria inhibit plant soil-borne diseases by producing antibiotics, secreting cell wall degrading enzymes, parasitism and inducing host resistance; meanwhile, Actinobacteria can also promote plant growth through production of plant growth regulators, nitrogen fixation, production of ferric carriers and dissolved mineral phosphates (Palaniyandi et al., 2013; Ling et al., 2022). There are abundant pathogen suppressor members in the Bacteroidetes that contribute prominently to plant health as well as soil organophosphate mineralization (Lidbury et al., 2021). Among the bacterial genera with the average abundance >0.5%, the relative abundance of *Streptomyces* belonging to Actinobacteria decreased with the continuous cropping year in both the AKS and the SHZ soils. *Streptomyces* are the most abundant and important Actinomycetes, which contribute to the maintenance of soil fertility and homeostasis to promote plant growth and to help plants resist biotic and abiotic stresses (Pang et al., 2022). The decrease in the relative abundance of these phyla and genera might be an important cause of continuous cropping obstacles. Interestingly, the relative abundance of *Sphingomonas* increased with continuous cropping years in both soils. Previous studies have shown that *Sphingomonas* can help environmental remediation, plant growth and resistance to abiotic stresses (Asaf et al., 2020), but our results indicate that it appears to promote continuous cropping obstacles, and its specific role in continuous cropping soil needs further investigation.

LEfSe analysis was used to find robust differential species, i.e., biomarkers, between continuous cropping years in the two soils. This study revealed that Propionibacterium, Nocardioidaceae, and Actinobacteria were simultaneously biomarkers of two types of soil in the early stage (1–3 years) of continuous cropping, suggesting that these groups may be important species to maintain soil health in the early stage of continuous cropping. At the same time, we found that some taxa were enriched in the AKS soil with 1–5 years of continuous cropping and also in the SHZ soil with 15–20 years of continuous cropping, which might be due to the differences in the two soils origin and the composition of the native microbial communities.

Network analysis has been widely applied to explore the complex inter relationships and co-occurrence patterns of soil microbial communities (Faust and Raes, 2012). Previous studies have shown that agricultural measures and environmental changes affect the complexity of the soil microbial co-occurrence network; for example, agricultural intensification reduces the complexity of the soil fungal microbial co-occurrence network (Banerjee et al., 2019); soil erosion also leads to a reduced complexity of the microbial network (Qiu et al., 2021). This study was the first to explore the effects of continuous cropping on the co-occurrence network of soil bacterial communities. The results showed that long-term continuous cropping decreased the complexity of bacterial community co-occurrence network in the AKS soil, but increased the complexity of bacterial community co-occurrence network in the SHZ soil. The different responses of the co-occurrence networks of two soil communities to long-term continuous cropping might be due to the different complexities of the initial network (short-term continuous cropping). In addition, a lower proportion of positive links in soil bacterial communities with long-term continuous cropping might mean less collaboration between OTUs.

## Conclusion

The results of this study indicated that continuous cropping and soil origin jointly affected the diversity and structural of the soil bacterial population. The relative abundance of some beneficial bacterial taxa in both soils decreased with increasing years of continuous cropping, such as: Actinobacteria, Bacteroidetes, *Streptomyces*, Propionibacteriales, and Nocardioidaceae, and these taxa that were lost due to the continuous cropping might be one of the causes for continuous cropping obstacles. These findings extend our understanding of the mechanisms by which continuous cropping of cotton affects soil microbes and provide useful guidance for overcoming continuous cropping obstacles through the application of beneficial microbes in the future.

## Data availability statement

The datasets presented in this study can be found in online repositories. The names of the repository/repositories and accession number(s) can be found below: NCBI Sequence Read Archive (https://www.ncbi.nlm.nih.gov/), SRP412954.

## Author contributions

FW and HZ planned and designed the research and experiments. ZM, PL, CY, ZF, HF, LZ, YZ, and JZ performed the experiments. FW and ZM analyzed the data. FW, ZM, and PL wrote the manuscript. FW acquired the funds for the study. All authors read and approved the final manuscript.

## Funding

This work was supported by the National Natural Science Foundation of China (grant number 31901938).

## Conflict of interest

The authors declare that the research was conducted in the absence of any commercial or financial relationships that could be construed as a potential conflict of interest.

## Publisher’s note

All claims expressed in this article are solely those of the authors and do not necessarily represent those of their affiliated organizations, or those of the publisher, the editors and the reviewers. Any product that may be evaluated in this article, or claim that may be made by its manufacturer, is not guaranteed or endorsed by the publisher.

## Footnotes

^1^ https://huttenhower.sph.harvard.edu/galaxy/

^2^ http://mem.rcees.ac.cn:8081/

^3^ https://www.chiplot.online/
