# Supplementary material for: Soil bacterial community response to continuous cropping of cotton
Source: Front Microbiol. 2023 Jan 26;14:1125564. doi: 10.3389/fmicb.2023.1125564 (PMC9909236; doi:10.3389/fmicb.2023.1125564)
Supplement: Supplementary file 1 [file Data_Sheet_1.docx]

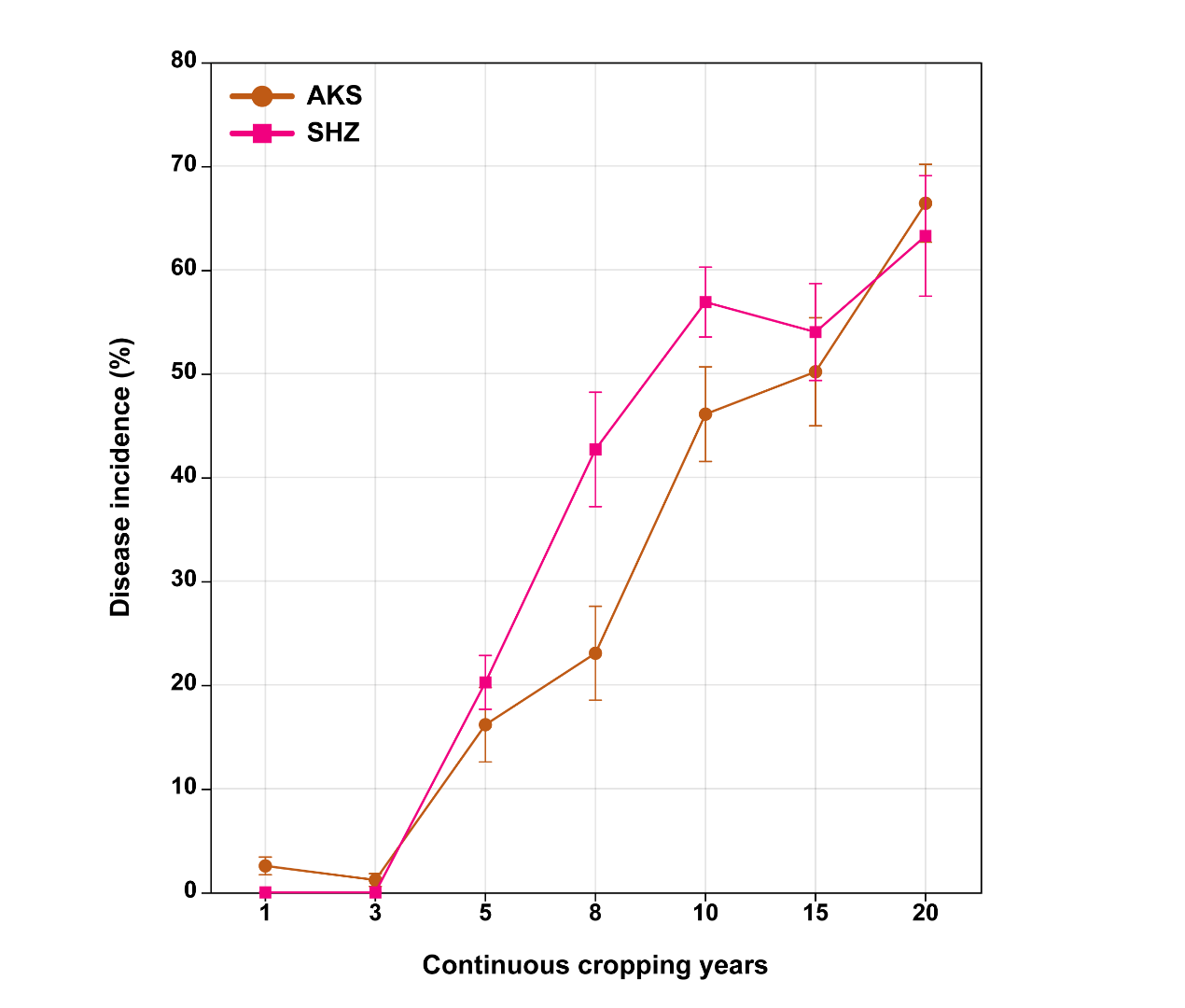


**Figure S1.** Incidence of Verticillium wilt in AKS and SHZ cotton fields in continuous cropping for 1 to 20 years.


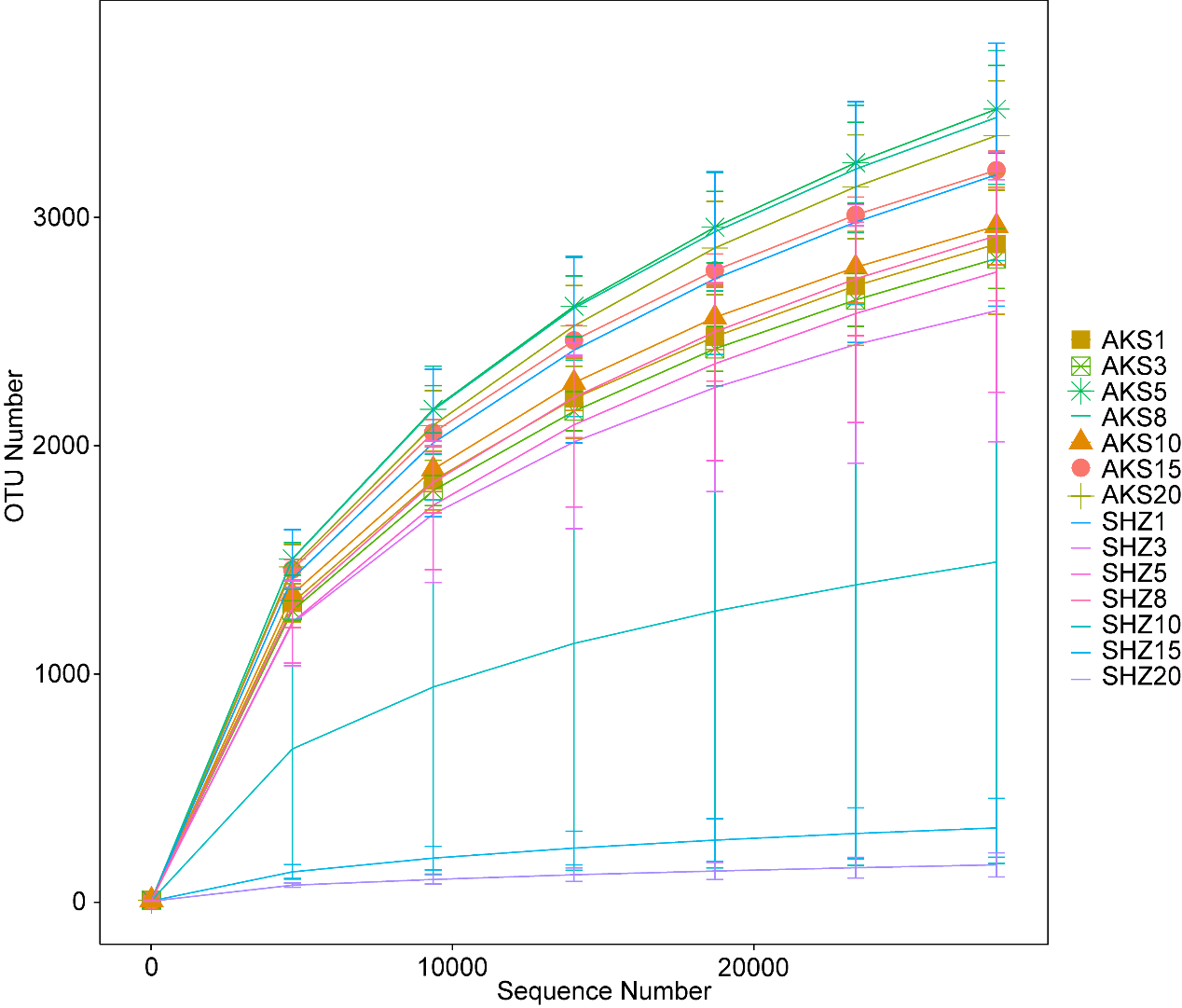


**Figure S2.** Rarefaction curves of 16S rRNA genes. The horizontal axis represents the number of sequences, and the vertical axis represents the number of observed species.


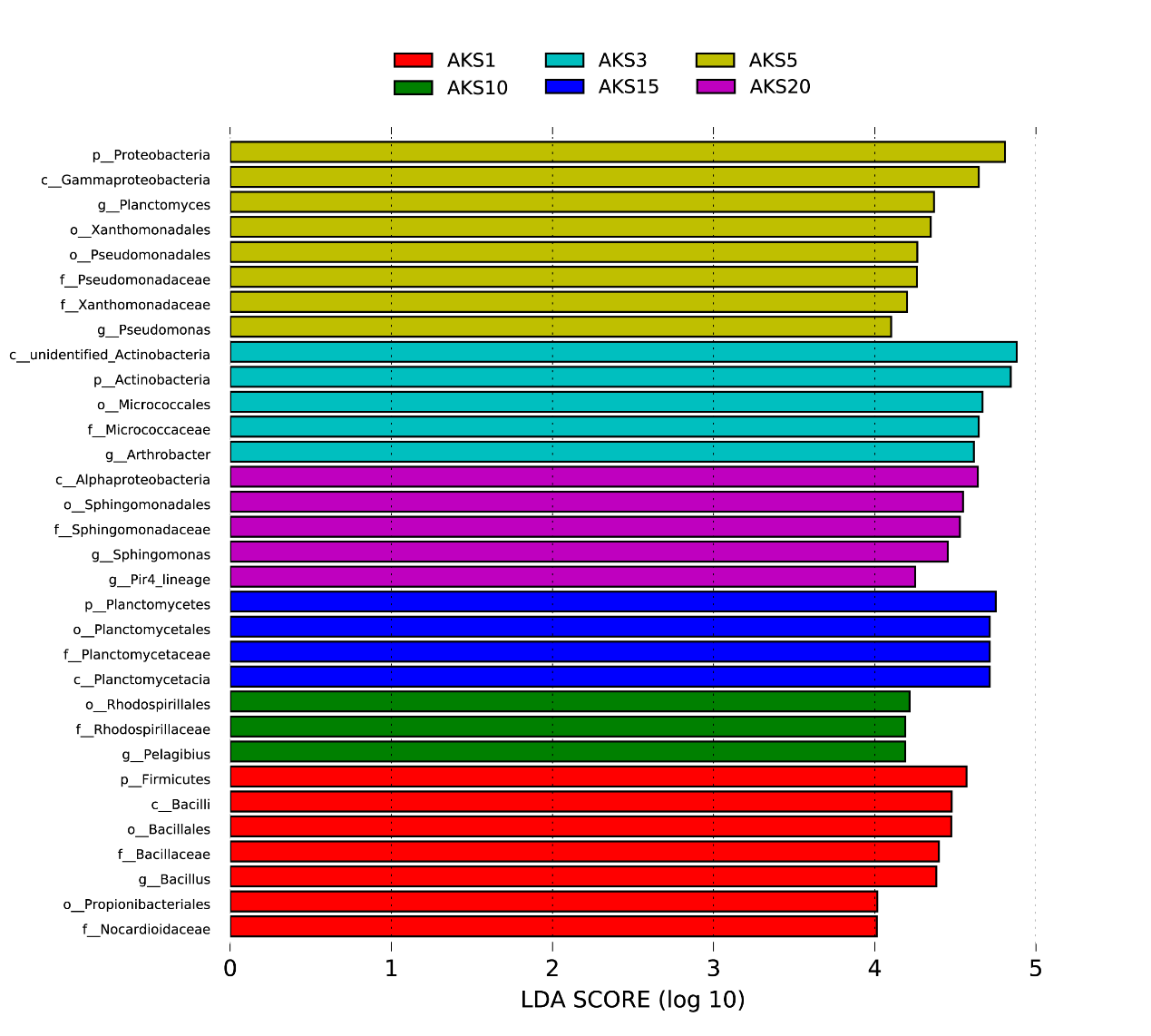


**Figure S3.** Results of LEfSe analysis showing bacterial taxa that differed significantly across the seven treatments in the AKS soil.


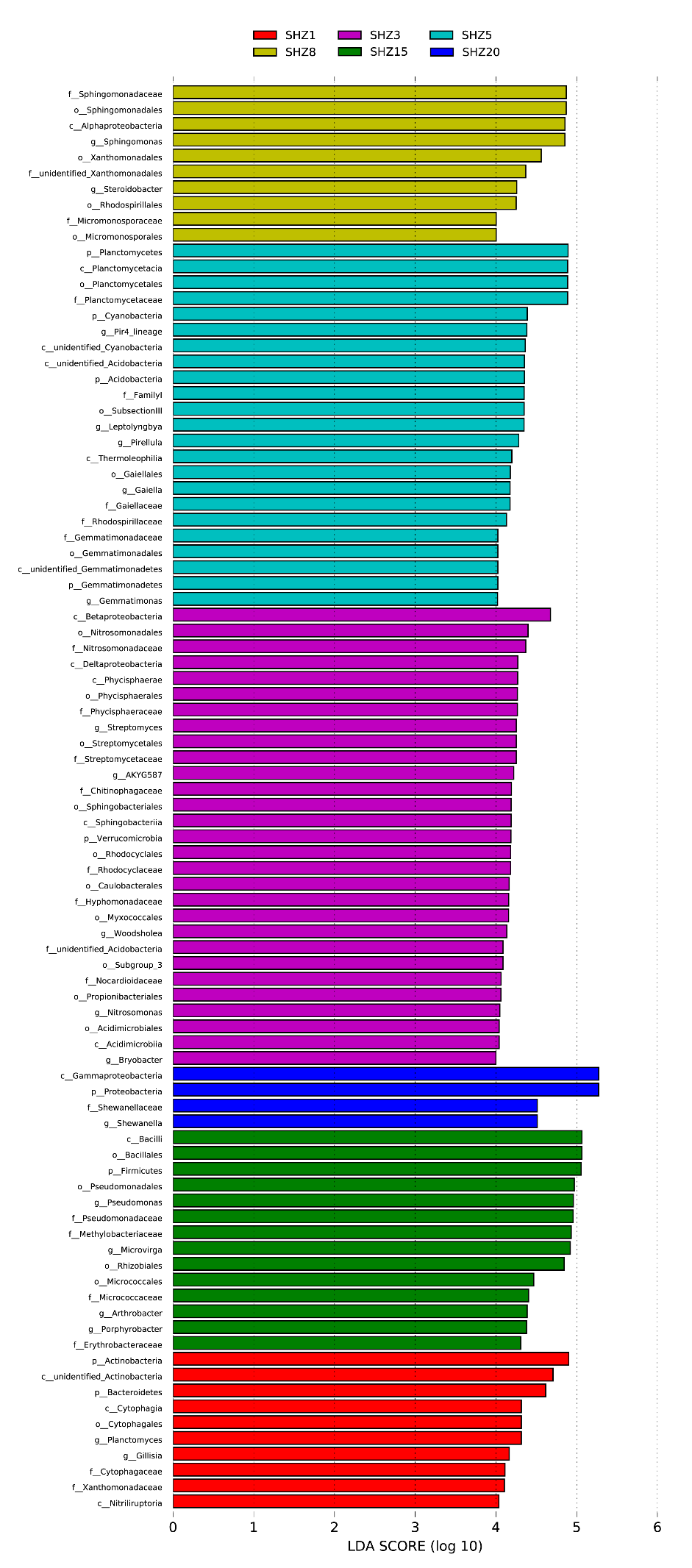


**Figure S4.** Results of LEfSe analysis showing bacterial taxa that differed significantly across the seven treatments in the SHZ soil.
